# Organizing everyday management in older adults with multimorbidity: a qualitative study from a time–geography perspective

**DOI:** 10.3389/fmed.2026.1837430

**Published:** 2026-06-18

**Authors:** Huiyu Lin, Huiling Lin, Zhishou Miao, Yiping Chen, Zhuo Wang, Xiaobing Wang

**Affiliations:** 1 Department of Nursing, Zhongshan Hospital, Xiamen University, Xiamen, Fujian, China; 2 School of Nursing, Beijing University of Chinese Medicine, Beijing, China; 3 Department of International Medical Services, Peking Union Medical College Hospital, Chinese Academy of Medical Sciences & Peking Union Medical College, Beijing, China; 4 Department of Pharmacy, Zhongshan Hospital, Xiamen University, Xiamen, Fujian, China

**Keywords:** multimorbidity, older adults, qualitative study, self-management, time-geography

## Abstract

**Background:**

Multimorbidity is common among older adults and is associated with substantial treatment burden, functional decline, and complex care needs. Although self-management has received increasing attention, less is known about how older adults organize everyday management activities within the temporal and spatial structures of daily life. This study aimed to explore how older adults with multimorbidity organized their everyday management activities from a time–geography perspective.

**Methods:**

A qualitative descriptive study was conducted with 26 community-dwelling older adults with two or more chronic conditions. Participants were purposively recruited through the outpatient pharmacy of a tertiary hospital. Semi-structured interviews were conducted face to face and focused on daily routines, management activities, bodily changes, healthcare use, and adjustment strategies. Interviews were audio-recorded, transcribed verbatim, and analyzed using thematic analysis informed by time-geography.

**Results:**

Data from 26 interviews reached saturation. Thematic analysis generated four themes and eight subthemes: (1) management embedded in everyday routines (management within household life, routine as a stabilizing strategy); (2) capability constraints and fragile routines (symptom fluctuation and disrupted plans, limited mobility, energy, and digital capacity); (3) coordination burden under coupling and authority constraints (constant alignment with people and facilities, restricted choice under rules and roles); and (4) active reorganization under multiple constraints (simplifying and planning ahead, unequal capacity to absorb disruption).

**Conclusion:**

Everyday management among older adults with multimorbidity is not a separate or clearly bounded medical activity but a dynamic time–space process embedded in daily life. A time–geography perspective helps reveal how personal, relational, and institutional constraints shape management practices and highlights the need for more realistic, person-centered support for older adults living with multimorbidity.

## Introduction

Multimorbidity is one of the most common health challenges among older adults and poses a major burden on healthcare systems worldwide (1). Compared with living with a single chronic condition, older adults with multimorbidity are more likely to experience polypharmacy, functional decline, greater healthcare utilization, and increasingly complex care needs (1, 2). As a result, research in recent years has shifted from a disease-specific focus toward a broader concern with how older adults cope with and manage multiple chronic conditions in everyday life (2, 3).

Existing evidence suggests that self-management in multimorbidity extends far beyond following medical advice. In daily life, older adults are often required to coordinate a wide range of activities, including medication use, symptom monitoring, healthcare appointments, diet, physical activity, and family responsibilities (4, 5). Qualitative studies have shown that patients often place greater value on maintaining a sense of normality, autonomy, and a manageable daily rhythm than on strictly meeting biomedical targets (6, 7). At the same time, when the workload associated with treatment, service demands, and polypharmacy exceeds what patients can realistically handle in everyday life, treatment burden accumulates and may further compromise their capacity for self-management (4, 5).

Despite growing attention to multimorbidity management, important gaps remain in the current literature. First, self-management has largely been framed as an individual behavioral issue, with emphasis placed on adherence, knowledge, and motivation, while less attention has been paid to how management activities are actually organized within the temporal and spatial structures of daily life (8). Second, although treatment burden and integrated care have increasingly been recognized as important, existing studies often describe barriers in isolation and pay limited attention to how physical limitations, service coordination, and institutional arrangements interact to shape patients’ everyday management practices (9). Third, recent research has highlighted the growing relevance of digital care, healthcare choices, and person-centered approaches for older adults with multimorbidity, while also indicating that patients’ capacity to act is deeply shaped by contextual conditions such as service accessibility, family support, and the availability of technology (10–13).

A time-geographical perspective offers a useful framework for addressing these limitations. Originally developed by Hägerstrand (14), time geography conceptualizes everyday life as trajectories unfolding across time and space, rather than as activities occurring in unrestricted contexts. Within this perspective, daily activities of older adults with multimorbidity are shaped by three forms of constraints: capability constraints, referring to limits arising from bodily conditions or personal resources, such as fatigue, pain, mobility problems, or limited digital skills; coupling constraints, referring to the need to coordinate activities with other people, transport, healthcare services, family responsibilities, and institutional schedules; and authority constraints, referring to rules and regulations embedded in systems and institutions, such as appointment procedures, prescription requirements, service availability, and hospital routines. Recent studies on travel time, care accessibility, and the lived temporal experiences of people with multimorbidity suggest that these constraints are highly relevant for understanding how health-related activities are organized in everyday life (15, 16).

Against this background, this study adopts a time-geographical perspective and uses spatiotemporal trajectory interviews to explore how older adults with multimorbidity organize their daily management activities. This study aims to examine how everyday management is organized as a time–space process in daily life, how it is shaped by capability, coupling, and authority constraints, and how older adults adjust their routines under these conditions. In this study, “everyday management” refers to the ways in which health-related activities are organized, negotiated, and integrated within the broader context of daily life, including household routines, social roles, and personal responsibilities. It therefore encompasses both illness-related tasks and the everyday activities within which these tasks are embedded.

## Methods

### Study design

This study adopted a qualitative descriptive design to explore how older adults with multimorbidity organize their daily management activities from a time-geographical perspective.

### Participants and recruitment

Participants were recruited purposively through the outpatient pharmacy of a tertiary hospital in Xiamen, an urban coastal city in southeastern China. Older adults with multimorbidity who routinely collected medications for long-term chronic disease management were identified as potential participants with the assistance of pharmacy staff, and were approached by the researcher for invitation.

Eligible participants were aged 60 years or older, had been diagnosed with two or more chronic conditions, were living in the community, had been continuously engaged in disease management, had basic communication ability, and were willing to participate. Those with severe cognitive impairment, acute severe illness, or marked communication difficulties were excluded. Variation in age, sex, chronic disease combinations, living arrangements, family support, and healthcare use was sought to enhance sample heterogeneity. Recruitment continued until data saturation was reached. “Engaged in disease management” was defined as having ongoing involvement in activities such as medication use, symptom monitoring, or regular healthcare visits.

### Data collection

Data were collected through semi-structured interviews. The interview guide was developed based on the time-geographical framework and relevant literature on multimorbidity management. Interviews focused on participants’ daily routines, management activities, changes in bodily condition, healthcare use, and strategies for adjusting daily order.

To operationalize the spatiotemporal trajectory interview, participants were invited to reconstruct a recent typical day from waking to bedtime. A simple timeline sheet was used to guide recall of activity sequence, approximate time points, locations, and movements between places. Participants were prompted to describe where management activities occurred, how they moved between home, community spaces, and healthcare settings, and whether these activities depended on family members, transport, or scheduled services. The timeline was used as an elicitation tool to capture temporal ordering, spatial movement, and coordination processes, rather than as standardized quantitative measures. When disruptions were mentioned, participants were further asked how plans changed and how subsequent activities were reorganized. Example questions included: “What is a typical day like for you?” “When do you usually take medication, monitor your condition, or deal with physical discomfort?” “Do hospital visits, medication collection, or examinations affect your plans for the day?” and “How do you adjust your activities when your physical condition changes?” Interviews were conducted in a quiet and convenient setting chosen by the participant, lasted approximately 30–60 min, and were audio-recorded with consent. Field notes were written after each interview to capture contextual and non-verbal information. The interview topic guide is provided in Supplementary material S2.

### Data analysis

Data were analyzed using thematic analysis (17). Interviews were transcribed verbatim and checked against the recordings for accuracy. The researchers read the transcripts repeatedly to gain familiarity with the data, then conducted open coding on content related to daily activity arrangements, bodily changes, healthcare processes, family support, institutional constraints, and coping strategies. Initial coding was conducted independently by two researchers, followed by regular discussions to compare interpretations and refine themes. Related codes were compared across participants and grouped into higher-level themes. Both inductive and deductive approaches were used. Themes were generated from the data, while the time-geographical framework was used to interpret how management activities were embedded in everyday time–space paths and shaped by capability, coupling, and authority constraints. While the time–geography framework informed interpretation, themes were developed inductively from participants’ accounts rather than imposed *a priori*. Data collection continued until no substantially new patterns relevant to the research questions were identified.

### Rigor

Rigor was ensured through several strategies (18). Credibility was enhanced through iterative comparison between codes, themes, and the original transcripts, as well as regular discussions among the research team. The research team had a background in nursing and qualitative research, and reflexive discussions were conducted to minimize potential bias. Dependability was supported by keeping coding records, analytic memos, and theme development documents. Confirmability was strengthened by grounding interpretations in participants’ own accounts. Transferability was promoted by providing descriptions of the study context, participant characteristics, and data collection process.

### Ethical considerations

This study was approved by the ethics committee of the researchers’ institution (Approval No. 2025-085). All participants were informed of the study purpose, confidentiality procedures, and their right to withdraw at any time. Written informed consent was obtained before participation. All data were anonymized, and participants could stop the interview or withdraw from the study at any stage without adverse consequences.

## Results

### Participant characteristics

Data saturation was reached after interviewing 26 participants. The sample included 14 women and 12 men, with most participants aged 70–79 years (n = 13). Most lived with a spouse (*n* = 12), while 8 lived alone. In terms of multimorbidity, 10 participants had three chronic conditions, and 7 had four or more. Regarding physical functioning, 11 described their condition as relatively stable, whereas 15 reported fluctuating or marked limitations. Only 9 participants could independently manage healthcare-related digital tasks; the remainder required partial or full assistance. Family support was reported as adequate by 13 participants, while 13 described it as intermittent or limited. The monthly frequency of medication collection or clinic visits ranged from 1 time (*n* = 7) to ≥4 times (*n* = 8). Details are presented in Table 1.

**Table 1:** Characteristics of participants (*n* = 26).

Characteristics	Classification	*n*	%
Sex	Female	14	54.0
	Male	12	46.2
Age group (years)	60–69	8	30.8
	70–79	13	50.0
	≥80	5	19.2
Living arrangement	Living alone	8	30.8
	Living with spouse	12	46.2
	Living with children/family	6	23.0
Number of chronic conditions	2	9	34.6
	3	10	38.5
	≥4	7	26.9
Self-rated physical functioning	Relatively stable	11	42.3
	Fluctuating limitations	10	38.5
	Marked limitations	5	19.2
Digital ability for healthcare tasks	Independent	9	34.6
	Partially dependent	10	38.5
	Fully dependent	7	26.9
Family support	Adequate	13	50.0
	Intermittent	8	30.8
	Limited	5	19.2
Frequency of medication collection /clinic visits	1 time/month	7	26.9
	2–3 times/month	11	42.3
	≥4 times/month	8	30.8

## Findings

Thematic analysis generated four themes and eight subthemes. The findings showed that daily management was not experienced as a separate health task, but was embedded in participants’ everyday time–space routines and continually shaped by capability constraints, coupling constraints, and authority constraints. The four themes were: management embedded in everyday routines; capability constraints and fragile routines; coordination burden under coupling and authority constraints; and active reorganization under multiple constraints (Table 2). The relationships between themes, subthemes, and the three types of time–geography constraints are summarized in Supplementary Table S1. Additionally, each theme reflects a dominant constraint pattern: Theme 2 primarily illustrates capability constraints, Theme 3 reflects coupling and authority constraints, and Theme 4 captures how these constraints interact in everyday reorganization.

**Table 2:** Themes, subthemes, and illustrative quotations.

Themes	Subthemes	Illustrative quotations
Theme 1. Management embedded in everyday routines	1.1 Management within household life	“Taking medicine, eating, doing a bit of housework—they are all mixed together.” (P2)
		“It is not like I just sit there and focus only on managing my illness.” (P8)
	1.2 Routine as a stabilizing strategy	“Once the timing is fixed, it is less easy to forget.” (P6)
		“If the pace of life gets disrupted, my body reacts immediately.” (P5)
Theme 2. Capability constraints and fragile routines	2.1 Symptom fluctuation and disrupted plans	“Sometimes the body just does not listen.” (P12)
		“Everything that comes afterward has to be postponed.” (P11)
	2.2 Limited mobility, energy, and digital capacity	“I really cannot manage those smartphone appointments.” (P4)
		“After all that trouble on the way there and back, I am completely worn out.” (P13)
Theme 3. Coordination burden under coupling and authority constraints	3.1 Constant alignment with people and facilities	“Going to the hospital is not just a matter of me deciding to go.” (P9)
		“It is this constant trying to fit all the times together that is the most exhausting part.” (P10)
	3.2 Restricted choice under rules and roles	“Some medicines cannot be prescribed for a long time… I still have to make the trip.” (P6)
		“Many things are not up to you.” (P12)
Theme 4. Active reorganization under multiple constraints	4.1 Simplifying and planning ahead	“Now I sort all the medicines in advance.” (P1)
		“If something can be done in the morning, I do it in the morning.” (P11)
	4.2 Unequal capacity to absorb disruption	“If one step does not work out, everything afterward falls apart.” (P13)
		“When someone helps you, things are not so difficult.” (P3)

### Theme 1: management embedded in everyday routines

In line with our definition, everyday management was understood as embedded within broader daily life activities rather than as a separate domain. This theme included two subthemes: management within household life and routine as a stabilizing strategy. It describes how illness management was interwoven with domestic life and sustained through repeated daily rhythms rather than carried out as a distinct task.

#### Subtheme 1.1: management within household life

Most participants did not describe managing illness as something done separately from daily life. Instead, taking medicine, monitoring symptoms, adjusting diet, and resting were commonly carried out alongside cooking, shopping, housework, and caring for family members. Management was therefore experienced as something “mixed in” with everyday living.

One participant said, “When I get up in the morning, I first sort out breakfast, then I take my medicine, and then I check what is missing at home. If I still need to go downstairs to buy vegetables, by the time I come back, it is almost time to start preparing lunch again.” (P3) Another similarly noted, “It is not like I just sit there and focus only on managing my illness. Meals need cooking, clothes need putting away, and sometimes I also have to give my wife a hand.” (P8) Some participants described this embeddedness more directly in everyday language: “Now I just carry on while keeping an eye on things. Taking medicine, eating, doing a bit of housework—they are all mixed together.” (P2) “Once the family gets busy, it is impossible to care only about yourself.” (P10) Similar accounts were also expressed by other participants: “You just get on with the day while also minding the body.” (P15); “It is all done in between things, not separately.” (P18); “You cannot put household matters aside just because you are unwell.” (P22).

#### Subtheme 1.2: routine as a stabilizing strategy

Although daily life was often busy and layered with competing demands, many participants tried to preserve order through fixed times, repeated sequences, and regular rhythms. Such regularity was not described as ease, but as a practical strategy for preventing life from becoming disorganized.

One participant explained, “I now take my medicine more or less at the same times—one round in the morning after getting up, another before bed at night. Once the timing is fixed, it is less easy to forget.” (P6) Another linked routine to stability: “After dinner I go downstairs for a short walk. I do not go far, just move around a bit. When every day is more or less like that, you feel steadier inside.” (P12) Participants also emphasized that bodily stability depended on regularity: “If the pace of life gets disrupted, my body reacts immediately, so I try to keep each day roughly the same.” (P5) “Some things cannot be done one way today and another way tomorrow… fixing things into a routine actually makes life easier.” (P1) Similar views were expressed by others: “If I do things at about the same time every day, I feel less flustered.” (P16); “Keeping a routine makes the day easier to manage.” (P19); “Once the order is set, the mind feels steadier too.” (P23); “If the rhythm stays the same, the day is less likely to go wrong.” (P26).

### Theme 2: capability constraints and fragile routines

This theme included two subthemes: symptom fluctuation and disrupted plans and limited mobility, energy, and digital capacity. It captures how established routines remained fragile because they were repeatedly interrupted by bodily instability and limited personal resources.

#### Subtheme 2.1: symptom fluctuation and disrupted plans

Many participants described how fatigue, dizziness, pain, poor sleep, and unstable physical conditions repeatedly interrupted their daily arrangements. Whether something could be done often depended less on prior planning than on whether the body was “cooperating” that day.

One participant said, “Sometimes I wake up in the morning feeling weak, and then I just do not want to go out. I may have planned to pick up medicine and take a walk on the way, but then I just let it go.” (P6) Another explained, “If the dizziness is bad, I have to sit down and recover first. Everything that comes afterward has to be postponed.” (P11) Pain similarly reshaped activity: “When my legs hurt, I do not even want to walk, let alone exercise. Even cooking feels like something I want to simplify.” (P4) One participant directly summarized this fragility: “It is not that I do not want to follow the plan. Sometimes the body just does not listen.” (P12) Comparable descriptions were also found in other interviews: “If I sleep badly, the next whole day feels thrown off.” (P17); “Some days you can manage, some days you cannot.” (P20); “When the body feels wrong, everything else has to move back.” (P24); “I may have planned it all, but that does not mean I can actually do it.” (P25).

#### Subtheme 2.2: limited mobility, energy, and digital capacity

Beyond symptom fluctuation, participants also described mobility problems, lack of stamina, difficulty standing in queues, and limited ability to use smartphones or online appointment systems. These constraints narrowed what could realistically be managed in time and space.

As one participant put it, “What I fear most now is queuing. If I stand for a while, my legs cannot bear it.” (P7) Another highlighted digital dependence: “I really cannot manage those smartphone appointments. I still have to wait for my children to help me.” (P4) The burden of travel was also evident: “It is not that I dare not go to the hospital alone. It is just that after all that trouble on the way there and back, I am completely worn out when I return.” (P13) Another participant contrasted current limitations with earlier life: “When I was younger, I could just go out whenever I wanted. Now it is not like that. First I have to think whether these legs and this energy can hold up.” (P8) Similar concerns were raised by others: “A single trip out now can use up the whole day’s energy.” (P14); “If nobody helps with the phone, I do not know how to get through the process.” (P18); “It is not unwillingness, it is that the body cannot keep up.” (P21); “Too many steps in one trip and I start worrying before I have even left home.” (P26).

### Theme 3: coordination burden under coupling and authority constraints

This theme included two subthemes: constant alignment with people and facilities and restricted choice under rules and roles. It describes how management activities depended not only on participants’ own effort, but also on repeated coordination with others, institutional schedules, and family expectations.

#### Subtheme 3.1: constant alignment with people and facilities

Follow-up visits, medication collection, and examinations were often described as requiring repeated coordination with family members, doctors’ clinic times, transport, and service facilities. For many participants, the burden of management lay not only in illness itself, but in having to constantly align different times and places.

One participant stated, “Going to the hospital is not just a matter of me deciding to go. I also have to see whether my child has time.” (P9) Another explained, “Doctors have their own clinic hours, and on my side I also have to see whether things at home can be arranged. It is not like I can go whenever I want.” (P5) Transport was another source of pressure: “If an examination is booked early, I have to get up even earlier to catch the bus. If there is any delay on the road, I get anxious the whole time.” (P3) One participant captured this burden directly: “Sometimes it is not the illness itself that is troublesome. It is this constant trying to fit all the times together that is the most exhausting part.” (P10) Similar accounts were also expressed by others: “You have to match the doctor’s time, the transport, and the family’s availability.” (P15); “If one part does not line up, the whole day is affected.” (P19); “Going for one thing often means arranging three or four things around it.” (P22); “It is all about timing and whether everything can connect properly.” (P25).

#### Subtheme 3.2: restricted choice under rules and roles

Participants also described how outpatient schedules, prescription limits, review cycles, and in-person requirements constrained their options. At the same time, family-role expectations—such as not wanting to trouble children or not leaving household matters undone—further restricted how they organized care.

One participant said, “Some medicines cannot be prescribed for a long time, so when the time comes, I still have to go again. Even if my body does not want to move, I still have to make the trip.” (P6) Another expressed limited autonomy within the system: “We just go according to how the hospital arranges it. There is not really much for us to choose.” (P1) Family obligations also shaped decisions: “I know I should go for review earlier, but I keep thinking my children are busy, so if it can be postponed a bit, I postpone it.” (P4) “It is not that I do not want to do things in the way that suits me best. It is just that many things are not up to you.” (P12) Similar tensions were seen in other accounts: “You cannot always arrange things according to your own convenience.” (P16); “There are times set by the hospital, and you just have to follow them.” (P20); “I try not to trouble the children unless I really have to.” (P23); “A lot of the time, the decision has already been made for you.” (P24).

### Theme 4: active reorganization under multiple constraints

This theme included two subthemes: simplifying and planning ahead and unequal capacity to absorb disruption. It shows that participants were not simply passive recipients of disruption, but actively adjusted routines to preserve a workable everyday order, although their capacity to do so differed greatly depending on available support.

#### Subtheme 4.1: simplifying and planning ahead

Many participants described practical strategies for reducing disruption, such as pre-sorting medicines, keeping healthcare days free from other obligations, arranging activities according to expected energy levels, and avoiding unnecessary trips. These strategies aimed not at ideal management, but at maintaining a manageable order in daily life.

One participant explained, “Now I sort all the medicines in advance, one compartment for each day, so that when it is time to take them I do not have to rummage through everything again.” (P1) Another described preserving healthcare days from overload: “If I have to go to the hospital that day, I do not arrange anything else. Otherwise, when I come back, everything feels chaotic.” (P5) Some participants also adjusted tasks according to their bodily rhythm: “If something can be done in the morning, I do it in the morning. By the afternoon I have no energy.” (P11) “Whatever can be cut down should be cut down a little. Do not pile everything into one day—older people cannot carry that much.” (P7) Similar adaptive strategies were mentioned by others: “I think through the whole route before going out now.” (P14); “You have to simplify where you can, otherwise the day becomes too much.” (P21); “Planning ahead is the only way not to get thrown off.” (P26).

#### Subtheme 4.2: unequal capacity to absorb disruption

Although many participants developed coping strategies, their ability to contain disruption differed substantially. Those living alone, with limited mobility, low digital capacity, weak family support, or frequent healthcare needs were more likely to experience one disturbance as affecting the whole day. By contrast, participants with stronger support could more often keep disruption localized.

One participant living alone said, “I live by myself. If one step does not work out, everything afterward falls apart as well, and there is nobody to pick things up for me.” (P13) Another emphasized the stabilizing role of a spouse: “My wife is still here, and she can help keep things in mind for me. So sometimes when I get thrown off, she can pull me back a bit.” (P14) A third contrasted supported and unsupported situations: “When someone helps you, things are not so difficult. If everything depends on yourself, then even a small problem can make you panic.” (P3) Digital support also mattered: “My daughter helps me with things on the phone. Otherwise, there are many procedures I really would not know how to get through.” (P4) Similar contrasts were evident in other interviews: “Going to the hospital with someone and going alone are completely different things.” (P18); “If nobody can step in, one small problem becomes a big one.” (P20); “Some people have someone to rely on nearby; that changes everything.” (P22); “Whether life stays in order often depends on whether support is there when you need it.” (P26).

## Discussion

This study extends existing multimorbidity research in two important ways. First, by applying a time–geography lens, it shifts attention from identifying what self-management involves to examining how it is organized as a time–space process embedded in everyday life. This perspective makes visible the temporal sequencing, spatial movement, and coordination work that are often overlooked in studies focusing primarily on individual behaviors. Second, the study demonstrates that capability, coupling, and authority constraints are not discrete or independent factors, but interact dynamically across situations. Rather than operating in isolation, these constraints accumulate and intersect in ways that can either destabilize or sustain daily management routines.

Our findings extend recent multimorbidity research by showing not only what kinds of burdens older adults face, but also how these burdens unfold across time and space in daily life. Recent qualitative syntheses have similarly argued that self-management among older adults with multimorbidity is best understood as a dynamic process of balancing illness demands with ordinary life rather than as simple adherence to clinical advice (2, 6). Likewise, treatment-burden research has emphasized that the work of living with multimorbidity includes not only medication-taking and service use, but also the time, coordination, and disruption imposed on everyday functioning (4).

Our findings showed that disease management was deeply embedded in everyday household life. Participants did not usually describe self-management as an independent activity; instead, medication-taking, symptom monitoring, and dietary adjustment were interwoven with cooking, shopping, housework, and family care. This finding is consistent with recent evidence showing that older adults with multimorbidity often prioritize preserving a sense of normal life, continuity, and autonomy over pursuing disease management in a purely biomedical way (2, 6). Breckner et al. also reported that self-management in multimorbidity is closely tied to activities of daily living and that patients often need support not only for medical decision-making, but also for the practical organization of everyday care work (19). Corbett et al. similarly found that older adults managing cancer together with other chronic conditions negotiated health-related workload in relation to available resources, motivation, and daily responsibilities, rather than treating illness work as a separate sphere (6). Our findings therefore reinforce the view that management routines are embedded in ordinary life and should be interpreted in relation to patients’ ongoing efforts to keep life workable and recognizable. More importantly, our findings show how this embeddedness is not merely a contextual backdrop, but an organizing principle that shapes when, where, and how management activities can take place.

The present study also found that maintaining routine functioned as an important stabilizing strategy. Participants repeatedly described fixed schedules, repeated sequences, and familiar rhythms as ways to keep both body and life from “falling into disorder.” This finding resonates with treatment burden theory and related work on normalization, which suggest that patients often try to fit treatment tasks into sustainable daily routines so that healthcare work becomes manageable rather than overwhelming (4, 20). A recent systematic review of the use of burden-of-treatment theory also emphasized that treatment work becomes bearable when it can be normalized within daily life, whereas disruptions in routine often increase perceived burden and reduce patient capacity (20). In this sense, the routines described in our study were not merely habits; they were practical devices for stabilizing daily life under conditions of ongoing complexity. From a time–geography perspective, such routines can be understood as strategies for reducing uncertainty by aligning bodily rhythms with temporal and spatial regularities.

Our findings highlighted the importance of capability constraints. Fluctuating symptoms, poor sleep, pain, fatigue, and limited stamina repeatedly disrupted participants’ plans and reduced what they could realistically do on a given day. This is highly consistent with previous studies showing that older adults with multimorbidity frequently experience unstable functional capacity, which affects medication management, physical activity, clinic attendance, and other self-management behaviors (4, 19). Recent work has similarly shown that self-management capacity is not fixed, but fluctuates with health status, confidence, and available resources (21). Our findings therefore support the argument that treatment workload cannot be understood separately from patients’ capacity to carry that workload. In practical terms, this means that even well-established management plans may remain fragile when bodily capability is unstable.

A related finding concerned digital capacity. Several participants reported difficulty using smartphone-based appointment systems, digital information, or remote coordination processes, and many remained dependent on adult children or others for these tasks. This aligns with growing evidence that digital exclusion is a major issue for older adults and may intensify care inequalities, especially when service systems increasingly assume digital access (22). A recent scoping review on digital exclusion in older adults identified lack of skills, confidence, usability, and support as key barriers, while reviews focused specifically on multimorbidity have similarly noted that digital tools may support coordination and self-management only when they are usable, low-burden, and appropriately tailored (12, 23, 24). Fjellså et al. further showed that older adults with multimorbidity may either gain control through eHealth-supported coordination or lose control when capacity and system support are insufficient (25). Our findings add to this literature by suggesting that digital difficulty should be understood as part of capability constraint, because it directly shapes what forms of management remain possible in everyday life. This highlights how digital capacity operates not only as an individual attribute but also as a condition that shapes access to and coordination within healthcare systems.

This study also demonstrated that daily management was strongly shaped by coupling constraints and authority constraints. Participants often described medical visits, medication collection, and examinations as activities that required repeated alignment with children’s availability, transport schedules, clinic hours, and service procedures. The burden was therefore not only in “having to go,” but in continuously aligning multiple people, places, and schedules under constrained conditions. From a time–geography perspective, this coordination work represents a central mechanism through which everyday management becomes demanding. This finding closely mirrors recent qualitative studies on multimorbidity navigation, which found that older adults’ ability to navigate healthcare is strongly influenced by autonomy, mobility, technological literacy, family support, and the degree of fragmentation in the health system (26, 27). Simpson et al. and Peeler et al. likewise showed that people with multimorbidity, caregivers, and professionals often recognize similar care goals, but differ in priorities and coordination needs, resulting in persistent communication and management difficulties (28, 29). Our findings, which is reflected in Theme 3, particularly in participants’ accounts of coordination burden and restricted choices, also echo recent literature on person-centered integrated care, which emphasizes that older adults value continuity, coordination, effective communication, and involvement, yet often experience services as fragmented and difficult to align with daily life (30, 31). These findings illustrate how coupling and authority constraints jointly structure access to care, rather than operating as separate domains.

The role of authority constraints was also prominent. Prescription rules, appointment cycles, and institutional procedures restricted patients’ flexibility, while family-role expectations such as “not bothering children” or “not leaving family matters undone” further narrowed their room for choice. These findings are consistent with recent reviews indicating that older adults with multimorbidity often make healthcare choices within constrained systems and relational obligations, rather than from a position of full autonomy (30). Thölking et al. showed that healthcare decision-making in multimorbidity is shaped by what choices are realistically possible within personal and structural circumstances, while Aarønes et al. emphasized the importance of trust, personal priorities, and meaningful assessment processes in multimorbidity care (13, 32). Taken together, these perspectives highlight that decision-making is both structurally constrained and relationally negotiated. In this study, these dynamics are reflected in participants’ emphasis on trusting healthcare arrangements and prioritizing routines that remain feasible within the constraints of everyday life.

Our findings also showed that older adults were not passive recipients of burden. Participants actively simplified tasks, planned ahead, rearranged activities according to energy levels, and protected certain parts of the day from overload. This supports previous literature describing self-management in multimorbidity as a process of constant negotiation and adaptation rather than simple compliance (2, 6, 19). However, our study also showed clear inequalities in the capacity to absorb disruption. Those living alone, with limited mobility, weak family support, or low digital literacy were more vulnerable to having a single disruption affect the whole day. This finding strongly aligns with the cumulative complexity perspective, which argues that outcomes depend on the balance between workload and capacity (4), and with recent studies showing that healthcare navigation and self-management become especially difficult when support resources are scarce (26, 27). It also resonates with current evidence that person-centered and integrated care models are most beneficial when they are responsive to individual capacity, social context, and continuity needs, rather than offering standardized disease-oriented solutions (33).

Taken together, the present study contributes to the multimorbidity literature in two main ways. First, it adds a time–geography lens to existing discussions of self-management and treatment burden by showing how management work is embedded in daily time–space routines. Second, it demonstrates that capability, coupling, and authority constraints are not isolated influences, but interact in ways that can either destabilize or sustain everyday order. Although recent reviews have called for more coordinated, continuous, and person-centered care for older adults with multimorbidity (33, 34), our findings suggest that such care will remain limited unless it also accounts for the ordinary temporal organization of people’s lives. In this respect, the study helps explain why older adults may appear “non-adherent” in clinical terms while, from their own perspective, they are already doing substantial invisible work to keep life manageable.

### Implications for practice and further research

Reducing coordination burden should be a clinical and service priority. Several practical strategies can be considered. First, healthcare providers could align follow-up visits, routine examinations, and prescription renewal on the same day where possible, to reduce repeated travel and fragmented care. Second, prescription renewal procedures could be simplified for clinically stable patients, for example by extending prescription duration or allowing community-level renewal. Third, for older adults with limited digital capacity, services could provide appointment assistance, simplified procedures, or non-digital alternatives. These measures may help reduce the amount of coordination work transferred to older adults and support more manageable everyday care.

### Strengths and limitations

A major strength of this study is its use of a time–geography perspective, which enabled a more process-oriented understanding of multimorbidity management in everyday life. In addition, the sample included variation in living arrangement, functional status, support availability, and healthcare use, which enriched the range of experiences captured. However, several limitations should be considered. The study was conducted in one tertiary hospital outpatient pharmacy in an urban Chinese setting, which may influence the transferability of the findings. In rural areas, older adults may face longer travel distances, fewer healthcare resources, and more limited digital infrastructure, potentially intensifying capability and coupling constraints. In other cities or healthcare systems, differences in appointment systems, prescription duration, and service organization may shape different patterns of coordination burden. In addition, variations in family roles and availability of community-based care may influence how management activities are organized in daily life. These contextual differences should be considered when interpreting and applying the findings.

## Conclusion

In conclusion, daily management among older adults with multimorbidity is not a separate medical task but a dynamic, time–space process embedded in everyday life. Participants’ ability to sustain management depended not only on motivation, but also on fluctuating bodily capacity, the need to coordinate with others and institutions, and the flexibility of support resources available to them. By making these everyday processes visible, this study provides a more grounded understanding of treatment burden and highlights the importance of designing care that is coordinated, flexible, and responsive to patients’ lived realities.

## Data Availability

The raw data supporting the conclusions of this article will be made available by the authors, without undue reservation.

## References

[1] Nicholson K Liu W Fitzpatrick D Hardacre KA Roberts S Salerno J . Prevalence of multimorbidity and polypharmacy among adults and older adults: a systematic review. Lancet Healthy Longev. (2024) 5:e287–96. doi: 10.1016/S2666-7568(24)00007-2, 38452787

[2] Tian J Wang HY Peng SH Tao YM Cao J Zhang XG. Experiences of older people with multimorbidity regarding self-management of diseases: a systematic review and qualitative meta-synthesis. Int J Nurs Pract. (2024) 30:e13289. doi: 10.1111/ijn.13289, 39075877

[3] Holland E Matthews K Macdonald S Ashworth M Laidlaw L Fraser SD . The impact of living with multiple long-term conditions (multimorbidity) on everyday life: a qualitative evidence synthesis. BMC Public Health. (2024) 24:3446. doi: 10.1186/s12889-024-20763-8, 39696210 PMC11654051

[4] Lee JE Lee J Shin R Oh O Lee KS. Treatment burden in multimorbidity: an integrative review. BMC Prim Care. (2024) 25:352. doi: 10.1186/s12875-024-02586-z, 39342121 PMC11438421

[5] Sadeghi H Mohammadi Shahbolaghi F Hosseini M Fallahi Khoshknab M Harouni GG. Factors associated with self-management in older adults with multiple chronic conditions: a qualitative study. Front Public Health. (2024) 12:1412832. doi: 10.3389/fpubh.2024.1412832, 39346598 PMC11429008

[6] Corbett T Lee K Cummings A Birch B Calman L Fenlon D . Self-management by older people living with cancer and multi-morbidity: a qualitative study. Support Care Cancer. (2022) 30:4823–33. doi: 10.1007/s00520-022-06892-z, 35147757 PMC8831683

[7] Ytterbrink Nordenskiöld K Sandlund C Kappelin C Norman K Wachtler C. “So I call myself healthy”: a qualitative study on health perceptions and healthcare experiences in older adults with multimorbidity. BMC Prim Care. (2025) 26:1–12. doi: 10.1186/s12875-025-03010-w, 41094678 PMC12522901

[8] Haase KR Hall S Sattar S Puts M Dillman C Fitch M . Living with cancer and multimorbidity: a qualitative study of self-management experiences of older adults with cancer. Eur J Oncol Nurs. (2021) 53:101982. doi: 10.1016/j.ejon.2021.101982, 34265510

[9] Wu J Xue E Huang S Fu Y Chen D Shao J . Facilitators and barriers of integrated care for older adults with multimorbidity: a descriptive qualitative study. Clin Interv Aging. (2023) 18:1973–83. doi: 10.2147/CIA.S436294, 38050622 PMC10693763

[10] Wu J Shao J Chen D Xue E Huang S Zhang H . Developing an integrated care conceptual framework for older adults with multimorbidity within China’s integrated delivery system. Age Ageing. (2025) 54:afaf060. doi: 10.1093/ageing/afaf060 40091182

[11] Yi M Hui Y Hu L Zhang W Wang Z. The experiences and perceptions of older adults with multimorbidity toward e-health care: a qualitative evidence synthesis. Telemed J E Health. (2024) 30:2527–44. doi: 10.1089/tmj.2024.0211 38920002

[12] Wang C Zhang W Zhao F Sun Q Li D Li X . Needs and experiences of older adults with multiple morbidities using health technologies for self-management: a systematic review and meta-synthesis. Worldviews Evid-Based Nurs. (2025) 22:e70030. doi: 10.1111/wvn.70030, 40387399

[13] Thölking TW Girard D Engels Y Schoon Y van Wijngaarden EJ. Considerations of older adults with multimorbidity when facing healthcare choices: a qualitative systematic review. Patient Educ Couns. (2025) 138:109194. doi: 10.1016/j.pec.2025.109194, 40493988

[14] Hägerstrand T. What about people in regional science? Papers Reg Sci. (1970) 24:7–21. doi: 10.1111/j.1435-5597.1970.tb01464.x

[15] Mseke EP Jessup B Barnett T. Impact of distance and/or travel time on healthcare service access in rural and remote areas: a scoping review. J Transp Health. (2024) 37:101819. doi: 10.1016/j.jth.2024.101819

[16] Cheng C Christensen M. Living with multimorbidity through time: a meta-synthesis of qualitative longitudinal evidence. Healthcare (Basel). (2024) 12:446. doi: 10.3390/healthcare12040446, 38391821 PMC10887575

[17] Clarke V Braun V. Thematic analysis. J Posit Psychol. (2017) 12:297–8. doi: 10.1080/17439760.2016.1262613

[18] Morse JM. Critical analysis of strategies for determining rigor in qualitative inquiry. Qual Health Res. (2015) 25:1212–22. doi: 10.1177/1049732315588501, 26184336

[19] Breckner A Roth C Glassen K Wensing M. Self-management perspectives of elderly patients with multimorbidity and practitioners: status, challenges and further support needed? BMC Fam Pract. (2021) 22:238. doi: 10.1186/s12875-021-01584-9, 34836506 PMC8624621

[20] Smyth RC Smith G Alexander E May CR Mair FS Gallacher KI. A systematic review of the use of burden of treatment theory. J Multimorb Comorb. (2025) 15:26335565251314828. doi: 10.1177/26335565251314828, 40352785 PMC12064904

[21] Fu Y Wu J Guo Z Shi Y Zhao B Yu J . Exploring self-management behavior profiles in patients with multimorbidity: a sequential, explanatory mixed-methods study. Clin Interv Aging. (2025) 20:1–17. doi: 10.2147/CIA.S488890, 39807400 PMC11725232

[22] Ge H Li J Hu H Feng T Wu X. Digital exclusion in older adults: a scoping review. Int J Nurs Stud. (2025) 168:105082. doi: 10.1016/j.ijnurstu.2025.105082, 40279791

[23] Endalamaw A Zewdie A Wolka E Assefa Y. A scoping review of digital health technologies in multimorbidity management: mechanisms, outcomes, challenges, and strategies. BMC Health Serv Res. (2025) 25:382. doi: 10.1186/s12913-025-12548-5, 40089752 PMC11909923

[24] Smith L Simpson G Holt S Dambha-Miller H. Digital applications to support self-management of multimorbidity: a scoping review. Int J Med Inform. (2025) 202:105988. doi: 10.1016/j.ijmedinf.2025.105988, 40424867

[25] Fjellså HMH Husebø AML Braut H Mikkelsen A Storm M. Older adults’ experiences with participation and eHealth in care coordination: qualitative interview study in a primary care setting. J Particip Med. (2023) 15:e47550. doi: 10.2196/47550, 37782538 PMC10580142

[26] Lee PSS Chew EAL Koh HL Quak SXE Ding YY Subramaniam M . How do older adults with multimorbidity navigate healthcare? A qualitative study in Singapore. BMC Prim Care. (2023) 24:239. doi: 10.1186/s12875-023-02195-2, 37957559 PMC10644451

[27] Ginman H Sitch M. Older adults’ experiences of navigating healthcare whilst living with multimorbidity. Psychol Health. (2025) 40:1532–50. doi: 10.1080/08870446.2024.2339327, 38693663

[28] Simpson G Morrison L Santer M Hijryana M Farmer A Dambha-Miller H. Perceptions and experiences of living with and providing care for multimorbidity: a qualitative interview study. J Multimorb Comorb. (2024) 14:26335565241240820. doi: 10.1177/26335565241240820, 38529048 PMC10962039

[29] Peeler A Nelson K Agrawalla V Badawi S Moore R Li D . Living with multimorbidity: a qualitative exploration of shared experiences of patients, family caregivers, and healthcare professionals in managing symptoms in the United States. J Adv Nurs. (2024) 80:2525–39. doi: 10.1111/jan.15998, 38197539 PMC11154012

[30] Murphy S McCance T White PJ. How do older people experience person-centred integrated care? An integrative review of the evidence. Int J Integr Care. (2025) 25:21. doi: 10.5334/ijic.9066, 41427083 PMC12716249

[31] Wilson G Hutchison JS. In pursuit of a person-centered approach to care delivery: a qualitative descriptive study of the patient experience of a long-term conditions clinic in general practice. Qual Health Res. (2025) 35:680–96. doi: 10.1177/10497323241272003 39326875

[32] Aarønes TR Taraldsen K Kvæl LAH. Relying on trust: a qualitative study on home-dwelling older adults’ experiences and preferences with multimorbidity management in municipal healthcare assessments. Health Expect. (2025) 28:e70445. doi: 10.1111/hex.70445, 40990165 PMC12457978

[33] Remers TEP Jeurissen PPT Coremans A Olde Rikkert MGM van Dulmen SA. Experiences and preliminary effects of the comprehensive chrOnic caRe outpatiEnt (CORE) clinic for patients with multimorbidity in the hospital setting. J Eval Clin Pract. (2024) 30:1361–72. doi: 10.1111/jep.14053, 39031802

[34] Wu J Zhang H Shao J Chen D Xue E Huang S . Healthcare for older adults with multimorbidity: a scoping review of reviews. Clin Interv Aging. (2023) 18:1723–35. doi: 10.2147/CIA.S425576, 37868094 PMC10588749

