# Supplementary material for: Organizing everyday management in older adults with multimorbidity: a qualitative study from a time–geography perspective
Source: Front Med (Lausanne). 2026 Jun 18;13:1837430. doi: 10.3389/fmed.2026.1837430 (PMC13322870; doi:10.3389/fmed.2026.1837430)
Supplement: Supplementary file 1 [file Data_Sheet_1.PDF]

Table S1 Mapping of time–geography constraints onto themes and subthemes

Themes	Subthemes	Main constraint(s) illustrated	Explanation
Management embedded in everyday routines	Management within household life	Coupling constraints	Management activities were embedded in household responsibilities, family roles, and domestic obligations, showing how illness work had to be coordinated with ordinary social and family expectations.
Management embedded in everyday routines	Routine as a stabilizing strategy	Capability constraints	Fixed times and repeated sequences helped participants compensate for memory concerns, bodily instability, and limited energy, thereby stabilizing daily management.
Capability constraints and fragile routines	Symptom fluctuation and disrupted plans	Capability constraints	Fatigue, dizziness, pain, poor sleep, and fluctuating bodily conditions directly limited what participants could do and disrupted planned activities.
Capability constraints and fragile routines	Limited mobility, energy, and digital capacity	Capability constraints	Reduced mobility, low stamina, and limited digital skills constrained participants' ability to reach services, use appointment systems, and coordinate

			healthcare tasks independently.
Coordination burden under coupling and authority constraints	Constant alignment with people and facilities	Coupling constraints	Clinic visits, examinations, medication collection, transport, and family availability had to be aligned across specific times and places.
Coordination burden under coupling and authority constraints	Restricted choice under rules and roles	Authority constraints; coupling constraints	Prescription limits, appointment rules, in-person requirements, and family-role expectations restricted participants' flexibility and autonomy in organizing care.
Active reorganization under multiple constraints	Simplifying and planning ahead	Capability constraints; coupling constraints; authority constraints	Participants adjusted medication routines, travel plans, and daily schedules in anticipation of bodily limitations, service arrangements, and institutional requirements.
Active reorganization under multiple constraints	Unequal capacity to absorb disruption	Capability constraints; coupling constraints; authority constraints	The consequences of disruption depended on the interaction between bodily capacity, family support, digital support, service access, and institutional flexibility.

Supplementary Material S2

Interview Topic Guide (Spatiotemporal Trajectory Interviews)

1. Opening and rapport building

Could you briefly tell me about yourself (e.g., age, living situation, daily life)?
How long have you been living with your current health conditions?

2. Daily spatiotemporal trajectory reconstruction
(Using a simple timeline sheet)

Can you walk me through a typical recent day, from when you wake up to when you go to bed?
What do you usually do in the morning, afternoon, and evening?
Where do these activities usually take place?
Do you usually stay at home, or do you go out? If yes, where do you go?

Prompts (temporal dimension):

Around what time do you usually do these activities?
Are these activities done at fixed times, or do they vary?

Prompts (spatial movement):

Do you need to travel between places? How do you usually get there?
Is going out easy or difficult for you?

3. Health-related management embedded in daily life

During your day, when do you usually take medication?
When do you monitor your condition or deal with physical discomfort?
How do these health-related activities fit into your daily routines?

Prompts:

Do you treat these activities as separate tasks, or are they mixed into your daily life?
Can you give an example?

4. Capability constraints

How does your physical condition affect what you can do during the day?
Are there days when your plans change because of how your body feels?

Prompts:

What happens when you feel tired, in pain, or unwell?

Are there things you would like to do but cannot?

Digital capacity:

Do you use smartphones or online systems for healthcare (e.g., appointments)?
Is this easy or difficult for you?

5. Coupling constraints
Do your daily activities depend on other people (e.g., family members)?
When you go to the hospital, do you need someone to accompany you?

Prompts:

Do you need to coordinate your time with others?
How easy or difficult is it to arrange these things?

Coordination with systems:

Do transport schedules or hospital hours affect your plans?
Have you experienced situations where things did not align?

Family and social roles:

Do family responsibilities affect how you manage your health?
Do you ever delay care because of family considerations?

6. Authority constraints
Are there rules or procedures in the healthcare system that affect your daily life?

Prompts:

For example, appointment systems, prescription duration, or follow-up requirements
Do you feel you have choices in how you arrange your care?

7. Disruptions and reorganization
Can you describe a time when your daily routine was disrupted?

Prompts:

What caused the disruption?
What happened afterward?

Reorganization:

How did you adjust your plans?
What did you prioritize or give up?
8. Strategies for maintaining daily order
How do you usually keep your daily life manageable?

Prompts:

Do you plan ahead?
Do you simplify tasks?
Do you arrange activities based on your energy levels?
9. Role of support and variation in capacity
Who helps you manage your health or daily life?

Prompts:

What difference does it make when someone helps you?
What happens when you have to manage everything on your own?

8. Closing questions
Is there anything else about your daily life and health management that you would like to share?
What do you find most difficult in managing your daily life?
